# Outcomes From Real-World Data on Intraoperative Electronic Radiotherapy for the Treatment of Early-Stage Breast Cancer: Long-Term Recurrence and Survival Outcomes From a Single Center

**DOI:** 10.1155/2024/6207762

**Published:** 2024-11-14

**Authors:** Dolores De la Mata, Bernardino Gabriel Santiago-Concha, Juan Enrique Bargalló-Rocha, Carlos Daniel Robles-Vidal, Daniella Gómez-Pue, Gerardo Castorena-Rojí, José Hinojosa-Gómez, Fabiola Flores-Vázquez, Mónika Blake-Cerda, Mario Enriquez-Barrera, Antonio Maffuz-Aziz

**Affiliations:** ^1^Department of Radiation Oncology, Centro Médico ABC, Mexico City, Mexico; ^2^Department of Oncology Surgery/Gynecology Surgery, Centro Médico ABC, Mexico City, Mexico

**Keywords:** IORT, Lumpectomy, Mastectomy-free, Overall survival, Targeted radiotherapy

## Abstract

**Purpose:** This study is aimed at investigating the 10-year outcomes of intraoperative radiotherapy (IORT) in Mexican women with early breast cancer (EBC) treated at the Centro Medico ABC, Mexico City.

**Methods:** A cohort study included women with early-stage invasive ductal carcinoma aged ≥ 45 years without prior oncologic treatment, tumor size ≤ 3.5 cm, cN0M0, positive hormone receptors, margins ≥ 2 mm, negative sentinel lymph nodes, and no extensive lymphovascular invasion. IORT was administered at 20 Gy for 20–30 min after a lumpectomy. Follow-up extended over 10 years and included clinical examinations every 6 months for the first 18 months, followed by annual mammograms and conventional examinations. Patients out of the criteria were excluded from this study because they were referred for additional surgery and/or whole-breast radiation therapy.

**Results:** The study involved 238 patients with an average age of 61.1 years. The mean tumor size was 12 mm, and the percentages of lymphatic invasion, positive hormone receptors, and HER2/neu overexpression were 12.6%, 90.8%, and 2.1%, respectively. The median follow-up was 66.6 months (range: 1–126 months), and the overall survival and mastectomy-free rate reached 95.7% and 90%, respectively. Thirteen patients showed side effects; four recurrences were recorded, of which 50% were out-field relapses. The 5-year Kaplan–Meier probability of local relapses, mastectomy-free, and overall survival reached 97.5%, 100%, and 98%, respectively.

**Conclusions:** This is the first 10-year report about the effect of IORT on Mexican women with EBC in the early stages. Strict adherence to the selection criteria in this study resulted in low rates of side effects, mortality, and local recurrences, demonstrating that IORT is an effective treatment alternative for patients with EBC. Studies with a longer follow-up period should be performed, as recurrences can occur in the long term.

## 1. Introduction

Breast cancer (BC) remains one of the most prevalent cancers worldwide despite efforts dedicated to awareness campaigns and detection policies, making it a global health problem. In Mexico, in 2022, 23,790 new cases and 7888 deaths linked to BC were recorded [1]. Early diagnosis and appropriate treatment offer reasonable rates of disease control. The conventional treatment is breast-conserving therapy, which involves breast-conserving surgery (BCS) followed by whole-breast radiation therapy (WBRT). Between the ‛80s and 2000, it was demonstrated that BCT and mastectomy had similar survival rates in the treatment of early breast cancer (EBC) [2, 3]. In recent years, some studies have demonstrated higher survival of women treated by BCT [4, 5]. However, around 9%–28% of women who have BCS do not finish the recommended course of irradiation or may not receive any irradiation at all. Reasons given include the age of the patient, radiation-induced toxicities, side effects, the cost of daily therapy, increased exposure to medical environments, commuting to and from the radiation center, and the general logistics of WBRT [6, 7]. Therefore, hypofractionated radiotherapy has emerged as an excellent option for treating eligible patients, allowing 15-session regimens to replace the traditional 30-session treatments and, more recently, offering a 5-session regimen with an additional boost [8].

In this sense, accelerated partial breast irradiation is a targeted form of radiation therapy used primarily for patients with EBC. It focuses on delivering radiation to the area around the tumor while minimizing exposure to surrounding healthy tissue. The three common types of APBI are brachytherapy, external beam radiation therapy, and intraoperative radiotherapy (IORT) [9]. The IORT is the only accelerated partial breast irradiation technique that enables the performance of BCS and radiotherapy in a single procedure. This specialized therapy can be used as a treatment for BC in selected groups, delivering a single dose of high radiation (usually 20 Gy) directly to the tumor bed, minimizing exposure to surrounding healthy tissues [10, 11]. The application of this technique has been strongly supported by studies like TARGIT-A, an international, prospective, randomized, noninferiority Phase 3 trial that involved the treatment of 1113 patients with IORT and 1119 with EBRT. This report shows a slight difference in recurrence rates between the patients who received IORT and those who received EBRT (1.2% vs. 0.95%) during 4 years of follow-up [12]. However, IORT can lead to a lower risk of long-term side effects, such as damage to the heart, lungs, or skin. Moreover, high overall survival, low local recurrences, and low mastectomy rates have been reported [10, 11, 13–15]. Since IORT is performed during surgery, it eliminates the need for additional hospital visits for radiation therapy; this may reduce the overall cost of treatment [16]. This reduction can reach 12% of the total treatment cost of women with EBC in the Metropolitan Area of Mexico City [17]. For these reasons, in 2013, Centro Medico ABC of Mexico City launched the IORT program using the Intrabeam device to offer this alternative to patients who lived far from the hospital or could have undergone and preferred this treatment option over EBRT. This study is aimed at evaluating the long-term outcomes of EBC patients treated with perioperative single-dose IORT (Intrabeam), with a focus on local recurrences and overall survival.

## 2. Patients and Methods

### 2.1. Study and Profile of the Patients

This single-institution study was conceived and executed at the Radio-Oncology Department of Centro Médico ABC of Mexico City, Mexico. Between January 2013 and December 2021, women older than 45 years with early-stage invasive ductal breast carcinoma were eligible to be part of the study. In addition to this, the inclusion criteria were diagnosis established by biopsy, tumor size ≤ 35 mm (diameter), cN0M0, positive hormone receptors, margins ≥ 2 mm, negative sentinel lymph nodes (SLNs), no extensive lymphovascular invasion, without previous oncological treatment, and feasible treatment of BCS. Multifocal lesions and positive SLNs were used as exclusion criteria. Criteria were established in a multidisciplinary oncology committee of Centro Médico ABC (Mexico City, Mexico). Each patient provided full written informed consent and needs to remain accessible for a minimum of 8 years for routine follow-up. Patients out of the inclusion criteria were referred for further surgery and/or WBRT; they were not included in this study.

### 2.2. Preoperative Protocol

An axillary ultrasound was performed on all patients. The examination of SLN was performed by conventional lymphoscintigraphy using technetium-99m. In 0.2 mL of saline solution, 5–10 MBq of technetium-99m-labeled particles (50–200 nm) of human colloidal albumin (Nanocoll, GE Healthcare, Milan, Italy) was injected subcutaneously if the tumor was superficial and peritumorally if it was deep. To correctly detect the SLN, anterior and anterior–oblique lymphoscintigraphic projections of the breast and axilla were taken 15–30 min after the radiotracer injection [18, 19].

### 2.3. Surgery and IORT

**Figure 1:**
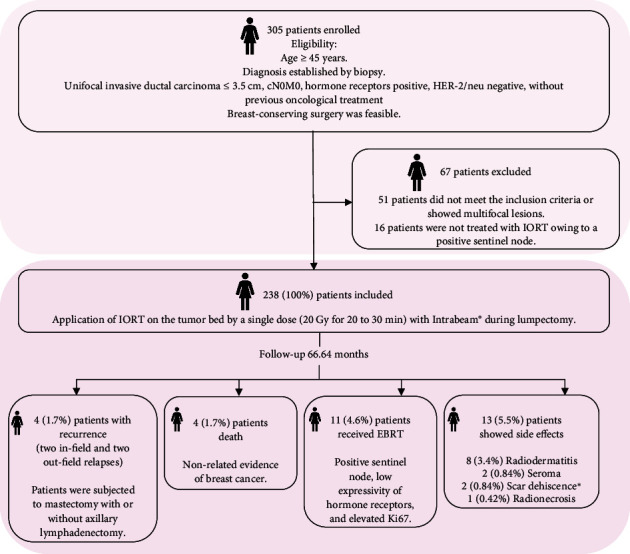
Recruitment flowchart for intraoperative radiotherapy (IORT) treatment and outcomes of follow-up for 66.64 months (range: 1–126 months) for 238 patients with early-stage breast cancer. EBRT: external breast radiation therapy.

The surgery was carried out 4–20 h after the tracer injection. A gamma ray–detecting probe in a sterile glove was used to perform macro and microscopical observations and confirm the absence of SLNs and free surgical margins. The margin was considered positive if the tumor was located at ≥ 2 mm of the inked surface [20]. Then, IORT was performed with an Intrabeam (Carl Zeiss Meditec, Oberkochen, Germany) device. To this spherical applicator, the size mainly used was 3.5 cm (range: 2–5 cm); the selection was performed by the surgeon and radiation oncologist and was attached to the breast parenchyma using a purse string to the surface of the applicator, and an ultrasound was used to verify that the applicator was completely attached to the tissue at a safe distance from the skin (> 10 mm). Then, a 20 Gy radiotherapy dose delivered from a source of 50 kV x-ray was administered for 20–30 min depending on the size of the applicator. The applicator was removed at the end of treatment, and the hemostasis was reviewed to complete the surgical procedure.  Figure 1 provides a summary of the trial profile.

### 2.4. Follow-Up and Evaluation of Outcomes

The follow-up was performed in all the patients by clinical exams every 6 months during the first 18 months. Then, annual follow-ups that included mammography and standard clinical examination were performed for the next years. Local recurrence was defined as a tumor occurring within 2 cm of the tumor bed. The remaining cases were categorized as distant metastases, axillary recurrence, or secondary BC. The patients received adjuvant endocrine therapy as indicated in a standard regimen (tamoxifen for premenopausal patients and letrozole or anastrozole for postmenopausal patients) for at least 5 years.

### 2.5. Evaluation of IORT's Side Effects

The Late Effects in Normal Tissues—Subjective, Objective, Management, and Analytic Scales (CTCAE V6.0) were used to assess the side effects of radiotherapy.

### 2.6. Statistical Analyses

Data analyses were performed with SPSS Version 25 (IBM, Armonk, NY, USA). Continuous and categorical variables were presented by mean ± standard deviation, number of patients, and their corresponding percentage *N*(%). The Kaplan–Meier plots for overall survival, mastectomy-free rate, and local recurrence were obtained in Excel using the add-in XLSTAT. The confidence of the data was assessed by the Greenwood method at 5%.

## 3. Results

### 3.1. Cohort Study

Between January 2013 and January 2024, 305 patients were recruited for the study. Of these, 51 patients were out of the inclusion criteria, and in 16 patients, the IORT was not an option owing to the presence of SLN metastatic disease. These patients were treated with other options like surgery and/or WBRT.

Therefore, 238 patients with an average age of 61.12 years (range: 45–85 years) underwent IORT after the lumpectomy. This cohort of patients was a low-risk group since the patients had an average tumor size of 12.02 mm (range: 0.2–35 mm), half of them were grade 2, whereas 2.1% and 15.3% showed elevated HER2/neu and Ki67, respectively. Patients who were HER2-positive received anti-HER2 therapy. The lymph node analysis revealed 90.9% of pN0 cases, 7.2% of pN1 micrometastases, and 1.9% of macrometastases. The characteristics of the patients and the tumor are shown in Table 1, corroborating that the patients fulfilled the inclusion criteria.

### 3.2. Outcomes of Lumpectomy and IORT

**Figure 2:**
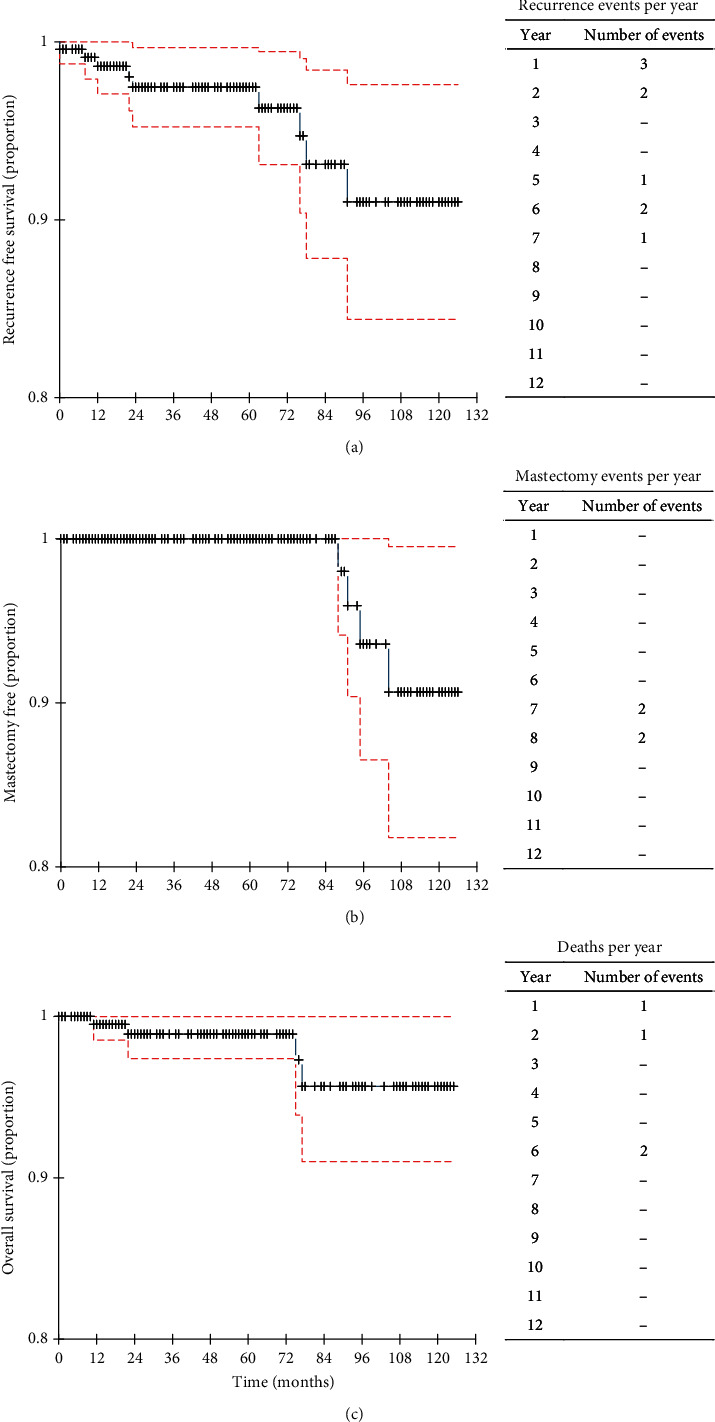
Cumulative proportions of (a) local recurrence–free, (b) mastectomy-free, and (c) overall survival of 238 patients with early-stage breast cancer treated by intraoperative radiotherapy with 10 years of follow-up. The dotted red lines represent the confidence intervals estimated by the Greenwood method at 5% confidence. Dashes (–) represent no events were recorded.

The 238 patients with EBC were treated with IORT and followed up for 66.64 months (range: 1–126 months). Four recurrences were recorded during this period. The local relapses (two patients) appeared after 77 months (range: 76–78 months) (Figure 2(a)). Patients with local recurrence underwent mastectomy with or without axillary lymphadenectomy. Two patients had recurrences outside the field; one of them was a multifocal recurrence that occurred 23 months after IORT treatment. The second recurrence occurred 12 months after surgery and treatment of the scar. The two patients who showed out-field relapses were younger than 50 years, which is related to the worst prognosis of invasive ductal carcinoma in premenopausal patients. Because of the limited number of events, the logistic regression found no correlation between the recurrences and the IORT treatment.

Four deaths were recorded during the follow-up period; none were related to BC (Figure 2(c)). Overall survival rates were exceptionally high, 98.91% and 95.66% at 5 and 10 years, respectively. In addition, the 5- and 10-year mastectomy-free rates were 100% and 90.51%, respectively, highlighting the effectiveness of the treatment in avoiding the need for this treatment (Figure 2(b)). These results underscore the favorable outcomes and low mortality rate associated with the treatment and highlight the effectiveness of the therapy in prolonging survival and maintaining breast health. The Kaplan–Meier probability for local relapses, mastectomy-free, and overall survival was 97.5%, 100%, and 98.0% at 5 years, whereas at 10 years, it was 90.9%, 90.7%, and 91.6%, respectively (Figure 2).

### 3.3. Side Effects and Additional Treatment

Thirteen (5.5%) patients showed adverse effects due to IORT; radiodermatitis was the most frequent side effect, followed by seroma, scar dehiscence (small scar dehiscence treated in consultation), and radionecrosis (Figure 1). Eleven (4.6%) patients received additional external radiation therapy for prevention, as the final pathology report indicated risk factors such as a positive sentinel node or low expressivity of hormone receptors (< 5%) and elevated Ki67 (> 15%) (Figure 1).

## 4. Discussion

BC is the most common type of cancer in women, which is often treated with lumpectomy and subsequent EBRT. An extra radiation dosage, known as a boost, is administered to the tumor bed if risk indicators are present [21]. To our knowledge, in Mexico, this is the report with the longer follow-up outcomes of IORT for treating BC in Mexican women. Previously, a retrospective study was conducted at the Instituto de Enfermedades de la Mama FUCAM, Mexico City, following the criteria of the American Society for Radiation Oncology and the European Society for Therapeutic Radiology and Oncology. The study included 230 patients with EBC at Stages I and IIA with a follow-up period of 48 months (range: 12–89 months). As a result, four (1.7%) local recurrences were registered, three of which were treated with breast conservation. In addition, 31 (13.5%) patients required EBRT, and 47 (23%) showed side effects (seroma, small dehiscence, edema, and mild radio epithelioid) that were treated in the medical office; the mastectomy-free rate was 96.96% [22]. All these outcomes are like those obtained in this report; however, the follow-up time in the current study is twice as long.

Previous studies reported shorter follow-ups with a lower number of patients. We obtained low rates of local recurrences, metastasis, and side effects, leading to an overall survival rate of 95.38% at 10 years. Our results agree with the data obtained in other world areas. This is comparable with the study of the Cleveland Clinic, which reported a 2% local recurrence rate in a cohort study of 201 patients with a 1.9-year follow-up [15]. The Rabin Medical Center in Israel and Beaumont University Hospital in the United States did not report local recurrences in follow-ups of 2.5 years in 158 patients [14] and 3.5 years in 61 patients [13], respectively. In both studies, the patients had tumors in Stage I or II. In the Catharina Hospital in The Netherlands, an 89.8% overall survival was reached after a 5-year follow-up in 306 patients [23]. However, compared with studies involving many patients, our results regarding recurrences and survival are significantly lower than those. In a single institution study conducted at Hoag Memorial Hospital Presbyterian, Newport Beach, California, United States, involving 1367 patients with 1400 different breast tumors, the estimated 5-year probability of local recurrence and overall survival rates was 5.27% and 96.3%, respectively [24]. This is like the ELIOT study performed at the European Institute of Oncology (Milan, Italy). In the study, electrons were used to deliver 21 Gy in a single dose to the tumor bed in 651 patients, compared to WBRT with normal fractionation (50 Gy in 25 fractions of 2 Gy plus 10 Gy boost) in 654 patients. After a median follow-up of 12.4 years, the 5-, 10-, and 15-year local recurrence rates for IORT were higher compared to those for patients treated with WBRT (4.2%, 8.1%, and 12.6% vs. 0.5%, 1.1%, and 2.4%, respectively). Although IORT had a significantly higher rate of local recurrence and axillary recurrence (1.9% vs. 0.3% for WBRT), there was no significant difference in distant disease, overall survival, or BC-specific survival. The ELIOT trial concluded that IORT should be offered to selected patients with a low risk of local recurrence, specifically those with a well-differentiated tumor ≤ 1 cm, Ki67 < 14%, and classified as luminal A. In that study, only 5% of ELIOT IORT patients (those with C4-positive nodes) got additional WBRT [25]. The selection criteria used in this study are close to the characteristics that describe a low risk of local recurrence in the ELIOT study. The results of the ELIOT study agree with the multicenter TARGIT-A study. The TARGIT-A 20 Gy was applied to 1140 IORT patients, whereas 1158 patients received WBRT (40–56 Gy, with or without a boost of 10–16 Gy). Local recurrence at 5 years in TARGIT-A study was 2.11% for IORT and 0.95% for WBRT. In the long term (maximum follow-up of 18.9 years, range: 7–10.6 years), there were no significant differences, for no statistically significant difference was found for local recurrence–free survival, mastectomy-free survival, distant disease-free survival, overall survival, and BC mortality between the assessed groups. Around 20% of IORT patients received WBRT [10]. It has been suggested that the need for additional treatment after IORT can be reduced by a better patient selection [24].

Local recurrences following IORT treatment for EBC are influenced by multiple factors. The most important is the adequacy of the surgical margins; if cancer cells are present at the margins of the removed tissue, the likelihood of recurrence increases [10, 11, 26]. In this study, only one patient showed positive margins; she was treated with additional WBRT. On the other hand, out-field recurrences after IORT treatment for EBC are primarily attributed to the presence of undetected microscopic cancer cells beyond the treated area, which were not targeted during surgery and can lead to recurrence in adjacent tissues [10, 27]. Biological characteristics of the tumor, such as aggressive subtypes or those with genetic mutations, as well as patient-related factors (young age and genetic predispositions), could also contribute to a higher likelihood of in- and out-field recurrence [10, 28]. In addition, the discrepancy between our study and others can be linked to the inclusion criteria. This report only involved patients with unifocal invasive ductal carcinoma; other studies also included patients with other histopathologic diagnoses like invasive lobular carcinoma, a combination of invasive lobular and ductal carcinoma, and even unknown diagnoses [10, 24, 29]. Invasive lobular carcinoma represents around 15% of BC cases; this type has a distinct biological behavior compared to ductal carcinoma and is generally harder to detect, often growing more diffusely, which may lead to a higher risk of recurrence [30]. Also, patients positive for SNL were involved [10, 24, 29]. Lymphovascular invasion and positive SNL are significant risk factors for recurrence and worse overall survival [31]. The discrepancy between the reported percentages of side effects after IORT treatment is particularly variable. Some of them, such as firm masses in the surgical cavity or other abnormalities, may go unreported by patients or may emerge long after treatment [32]. Future studies with standardized follow-up protocols and well-defined criteria for reporting late-onset effects are essential for accurately assessing the incidence of side effects.

The outcomes obtained in single-institution studies agree with the TARGIT-IORT. This randomized multicenter study (32 locations spread throughout 10 nations, including the United Kingdom, Europe, Australia, the United States, and Canada) involved 2298 patients, of whom 1140 underwent IORT and 1158 underwent EBRT. In the long-term follow-up (median 8.6 years, maximum 18.90 years), no statistically significant difference was observed in terms of BC mortality, overall survival, distant disease-free survival, local recurrence–free survival, or mastectomy-free survival. However, with TARGIT-IORT, deaths not related to BC were significantly lower than those with EBRT [10]. Therefore, IORT using low-energy x-rays (20 Gy) has been proven to be a safe and effective technique for EBC treatment [10, 12, 21].

Regarding the side effects of IORT, no risk variables were significantly associated with these events in this study. However, a correlation was suspected between the occurrence of side effects and the applicator size used in lumpectomy, which is related to the radiation dose [12, 29, 33]. In other reports, the presence of fat necrosis or seroma has been higher (11%) than that obtained in this study (0.84%) [14]. The variation in percentages and the occurrence of different side effects are influenced by multiple factors, including extensive tissue manipulation during surgery, high radiation doses, lymphatic disruption, inflammatory responses, tumor size and location, postoperative care, and patient-specific factors such as age and obesity [33–35].

IORT remains a contentious treatment, with critics arguing that the follow-up period of the TARGIT-A trial [12] is too short to conclude long-term local recurrence rates. However, TARGIT-IORT has demonstrated a low local recurrence rate of 2.5% [10]. Even this, there is a concern that radiation doses may be adequate to postpone but not prevent local recurrence. IORT has demonstrated favorable oncological outcomes in numerous clinical studies [8, 10, 12–15], including those with more flexible selection criteria. In Taiwan, a multicenter study across 26 hospitals treated 261 patients with IORT, with a mean age of 52.9 ± 9.8 years (range: 37–72 years) and larger tumors (mean size: 1.5 ± 0.8 cm, range: 0.1–4.2 cm) [35] compared to those in TARGIT-A [12] and TARGIT-IORT [10]. After an average follow-up of 15.6 months, two (0.8%) of the patients experienced locoregional recurrence, deemed an acceptable outcome [35]. We found that after 126 months of follow-up, low local recurrences, mortality linked to BC, and high mastectomy-free percentages can be reached in patients who met the eligibility criteria for IORT. Further long-term multistudy centers in Mexico and Latin America should be performed to guarantee these long-term outcomes.

## 5. Conclusions

This study provides the first 10-year report on the efficacy of IORT in Mexican women with EBC, focusing on those with unifocal invasive ductal carcinoma ≤ 35 mm and negative SNL. With a median follow-up of 66.6 months, the overall survival and mastectomy-free rates were 95.66% and 90.65%, respectively; this demonstrates that IORT is an effective treatment option, with low rates of adverse events, mortality, and local recurrence. These outcomes are consistent with global studies, reinforcing IORT as a safe and effective alternative for EBC, especially in well-selected patients. The strict selection criteria for the patient population in this study likely contributed to better outcomes compared to studies that included a wider range of histopathological types and additional unfavorable prognostic factors. IORT offers faster and more efficient treatment and helps reduce the burden on radiotherapy centers with long waiting lists. Nonetheless, further extensive multicenter studies across Mexico and Latin America are required to validate these promising long-term outcomes in a broader population.

## Figures and Tables

**Table 1:** Patient and tumor characteristics in the IORT.

**Characteristics**	**Patients (** **n** = 238**)**
Age (years)	61.2 (±10.07)
Tumor size (mm)	12.02 (±5.5)
Grade differentiation	
1	91 (38.2%)
2	120 (50.4%)
3	27 (11.4%)
Lymphovascular invasion	
Negative	174 (73.1%)
Positive	30 (12.6%)
Not reported	34 (14.3%)
Perineural invasion	
Negative	162 (68.1%)
Positive	21 (8.8%%)
Not reported	55 (23.1%)
Estrogen receptor	
Positive	218 (91.6%)
Negative	8 (3.4%)
Not reported	12 (5%)
Progesterone receptor	
Positive	216 (90.8%)
Negative	11 (4.6%)
Not reported	11 (4.6%)
HER2/neu overexpression	5 (2.1%)
Ki67 overexpression	36 (15.3%)

Data are the number of patients (percentages estimated based on the total included patients, *n* = 238).

Abbreviation: IORT, intraoperative electronic radiotherapy.

## Data Availability

Data are available on request from the authors.

## Nomenclature

BC: breast cancer
BCS: breast-conserving surgery
EBC: early breast cancer
IORT: intraoperative radiotherapy
WBRT: whole-breast radiation therapy
